# Supplementary material for: Molecular basis of bacterial DSR2 anti-phage defense and viral immune evasion
Source: Nat Commun. 2024 May 10;15:3954. doi: 10.1038/s41467-024-48291-4 (PMC11087589; doi:10.1038/s41467-024-48291-4)
Supplement: Supplementary file 1 — Supplementary Information [file 41467_2024_48291_MOESM1_ESM.pdf]

Supplementary Information

Molecular basis of bacterial DSR2 anti-phage defense and viral immune evasion

Jiafeng Huang[1,2,5*], Keli Zhu[1,5], Yina Gao[2], Feng Ye[1], Zhaolong Li[2], Yao Ge[1], Songqing Liu[2], Jing Yang[3*], Ang Gao[1,4,6*]

[1] Key Laboratory of Molecular Medicine and Biotherapy, Aerospace Center Hospital, School of Life Science, Beijing Institute of Technology, Beijing 100081, China

[2] Key Laboratory of Biomacromolecules (CAS), National Laboratory of Biomacromolecules, CAS Center for Excellence in Biomacromolecules, Institute of Biophysics, Chinese Academy of Sciences, Beijing 100101, China

[3] Department of Neurology, Aerospace Center Hospital; Peking University Aerospace School of Clinical Medicine, Beijing 100049, China.

[4] Science and Technology Innovation Center, Shandong First Medical University & Shandong Academy of Medical Sciences, Jinan, China.

[5] These authors contributed equally to this work.

[6] Lead contact

[*] Correspondence: jfhuang@ibp.ac.cn; yangjing@asch.net.cn; ang.gao@bit.edu.cn

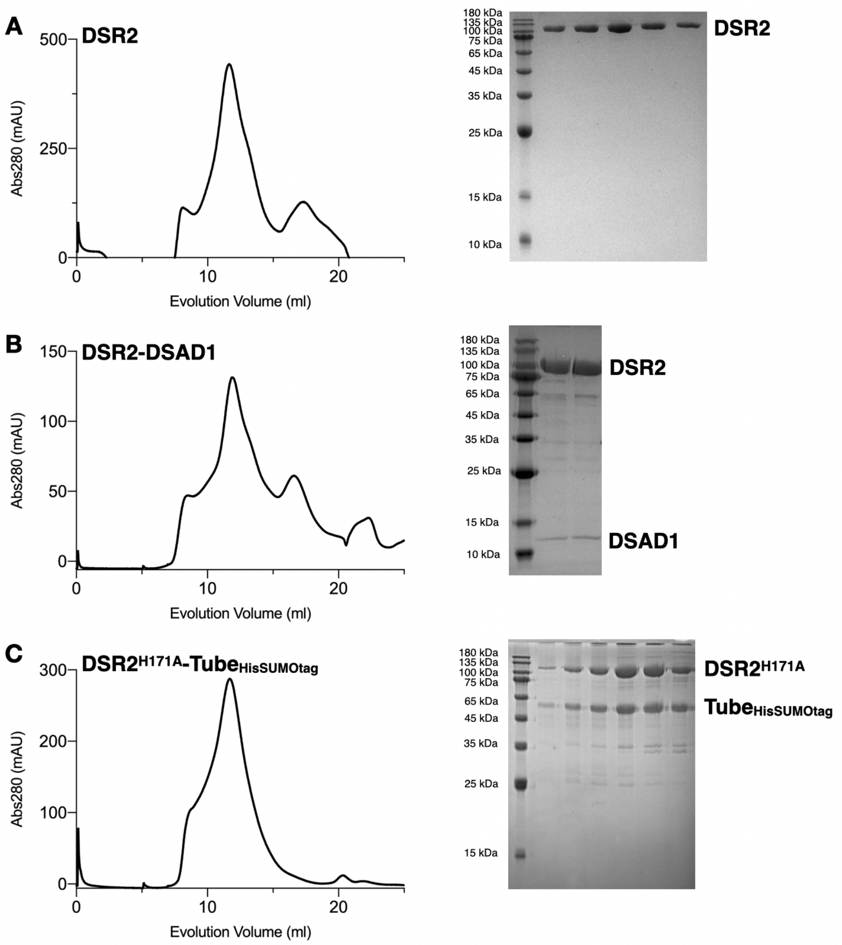

Supplementary Figure 1. Biochemical characterization of DSR2, DSR2-DSAD1 and DSR2^{H171A}-Tube$_{HisSUMOtag}$. A. Left, size exclusion chromatography analysis (Superdex 200 Increase 10/300 GL) of DSR2. Right, SDS-PAGE analysis of peak collections. **B.** Left, size exclusion chromatography analysis of DSR2-DSAD1, co-expression was used to obtain the complex. Right, SDS-PAGE analysis of peak collections. **C.** Left, size exclusion chromatography analysis of DSR2^{H171A}-Tube$_{HisSUMOtag}$. Right, SDS-PAGE analysis of peak collections.

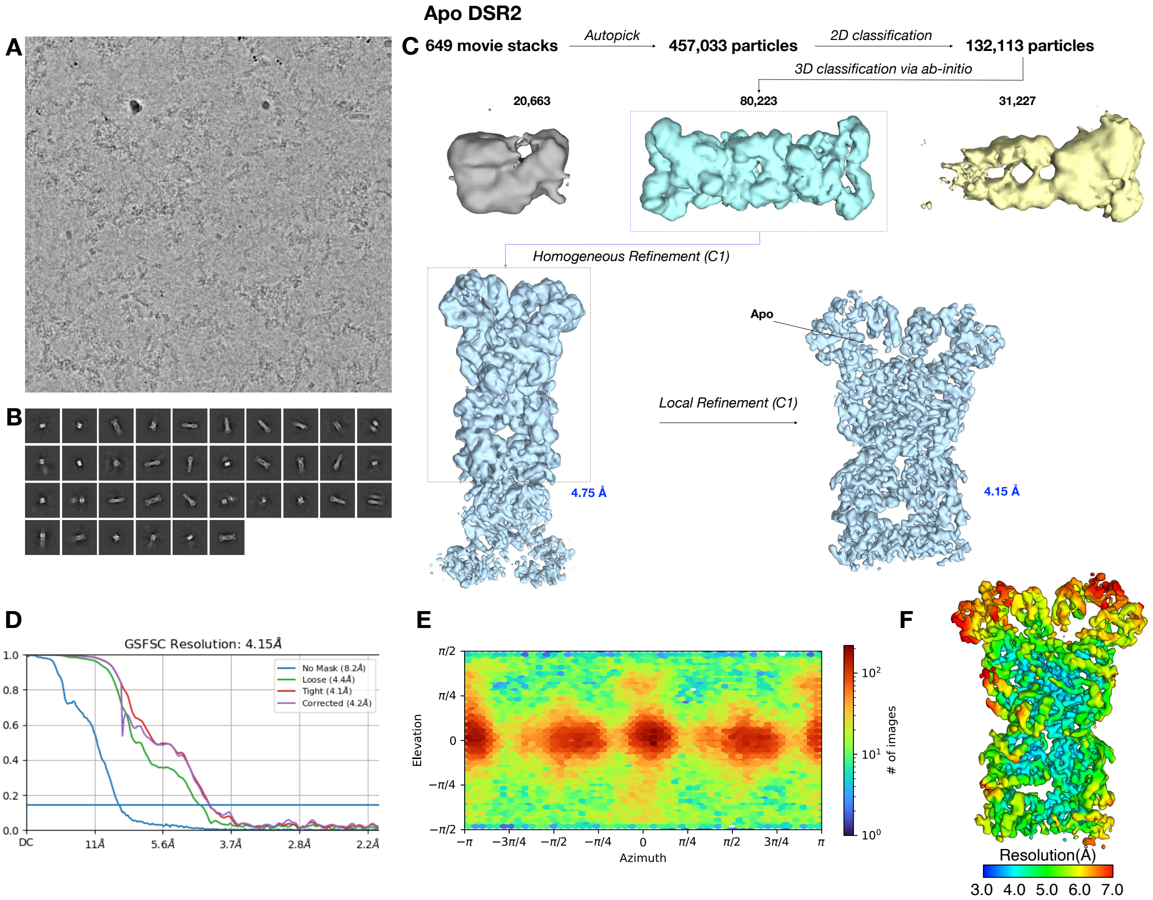

Supplementary Figure 2. Single particle cryo-EM analysis of the apo DSR2 tetramer. A. Representative cryo-EM micrograph of apo DSR2 tetramer. **B.** Representative reference-free 2D class averages of apo DSR2 tetramer. **C.** Data processing workflow for apo DSR2 tetramer. Gold standard Fourier Shell Correlation (FSC) curves for the reconstruction. **D.** FSC of unmasked and masked map (blue and red lines, respectively) are shown with resolutions at FSC = 0.143. **E.** Direction distribution plot of apo DSR2 tetramer. **F.** The maps colored to local resolution.

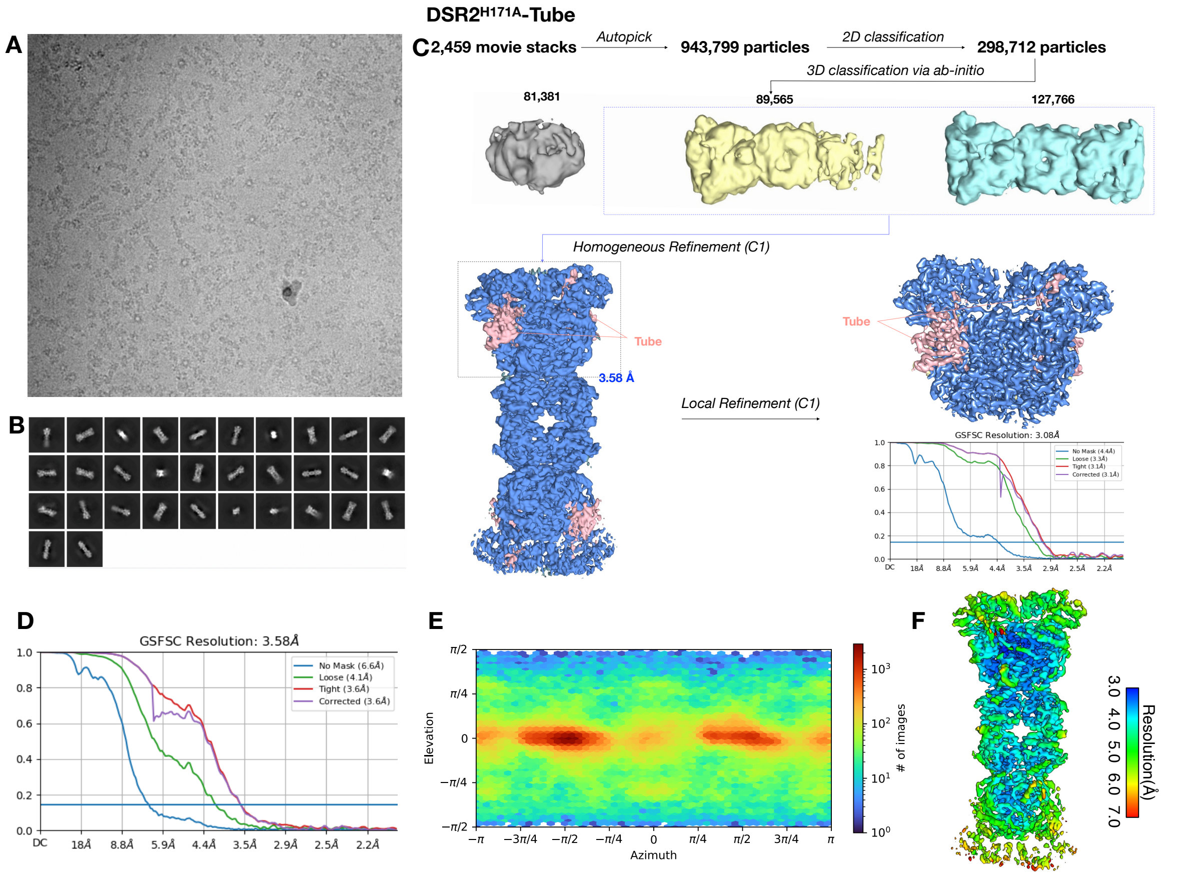

Supplementary Figure 3. Single particle cryo-EM analysis of DSR2^{H171A}-Tube. A. Representative cryo-EM micrograph of DSR2^{H171A}-Tube. **B.** Representative reference-free 2D class averages of DSR2^{H171A}-Tube. **C.** Data processing workflow for DSR2^{H171A}-Tube. Gold standard Fourier Shell Correlation (FSC) curves for the reconstruction. **D.** FSC of the unmasked and masked map (blue and red lines, respectively) are shown with resolutions at FSC = 0.143. **E.** Direction distribution plot of DSR2^{H171A}-Tube. **F.** The maps colored to local resolution.

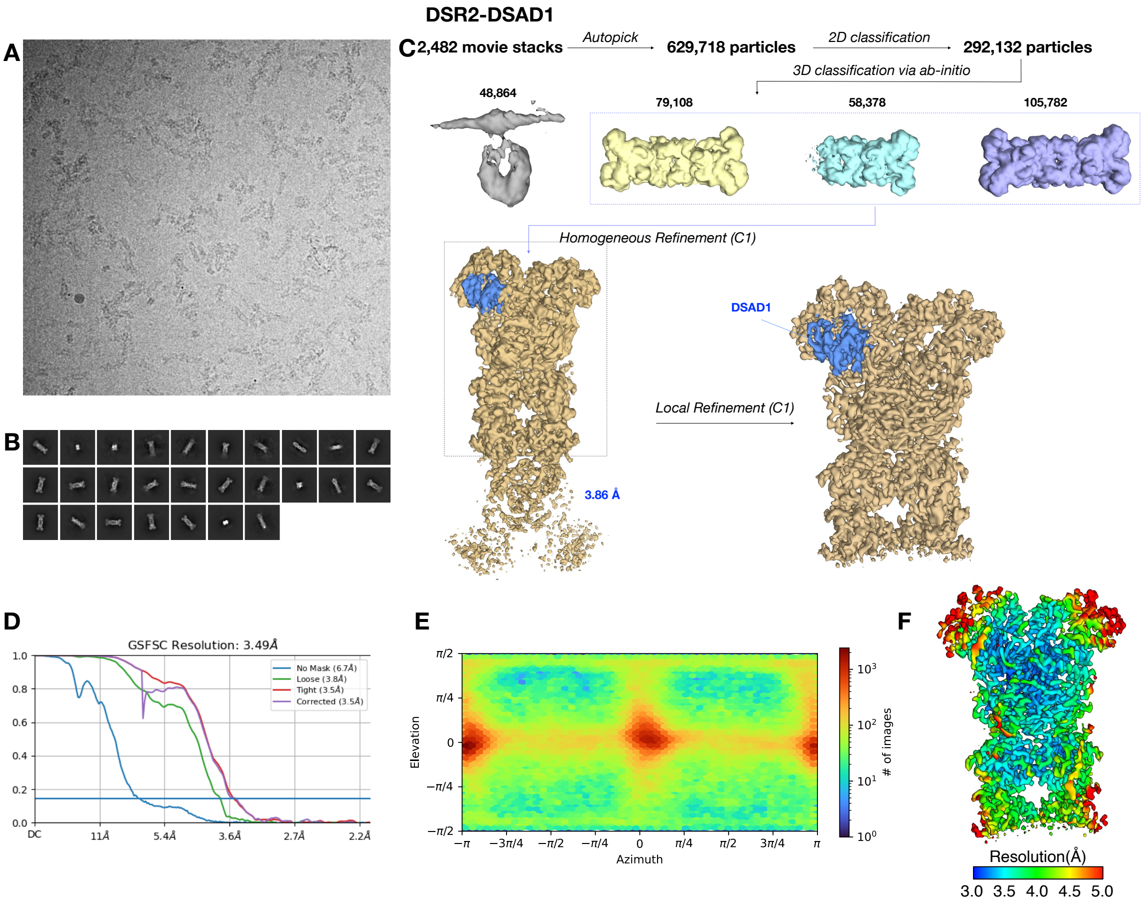

Supplementary Figure 4. Single particle cryo-EM analysis of DSR2-DSAD1. A. Representative cryo-EM micrograph of DSR2-DSAD1. **B.** Representative reference-free 2D class averages of DSR2-DSAD1. **C.** Data processing workflow for DSR2-DSAD1. Gold standard Fourier Shell Correlation (FSC) curves for the reconstruction. **D.** FSC of the unmasked and masked map (blue and red lines, respectively) are shown with resolutions at FSC = 0.143. **E.** Direction distribution plot of DSR2-DSAD1. **F.** The maps colored to local resolution.

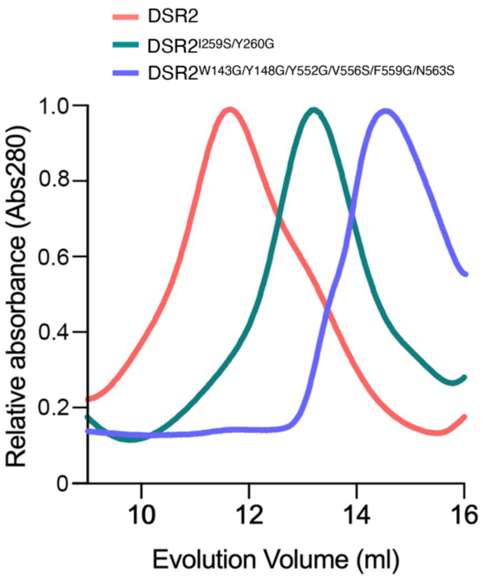

Supplementary Figure 5. Size-exclusion chromatography of wild-type DSR2, DSR2$^{I259S/Y260G}$ and DSR2$^{W143G/Y148G/Y552G/V556S/F559G/N563S}$.

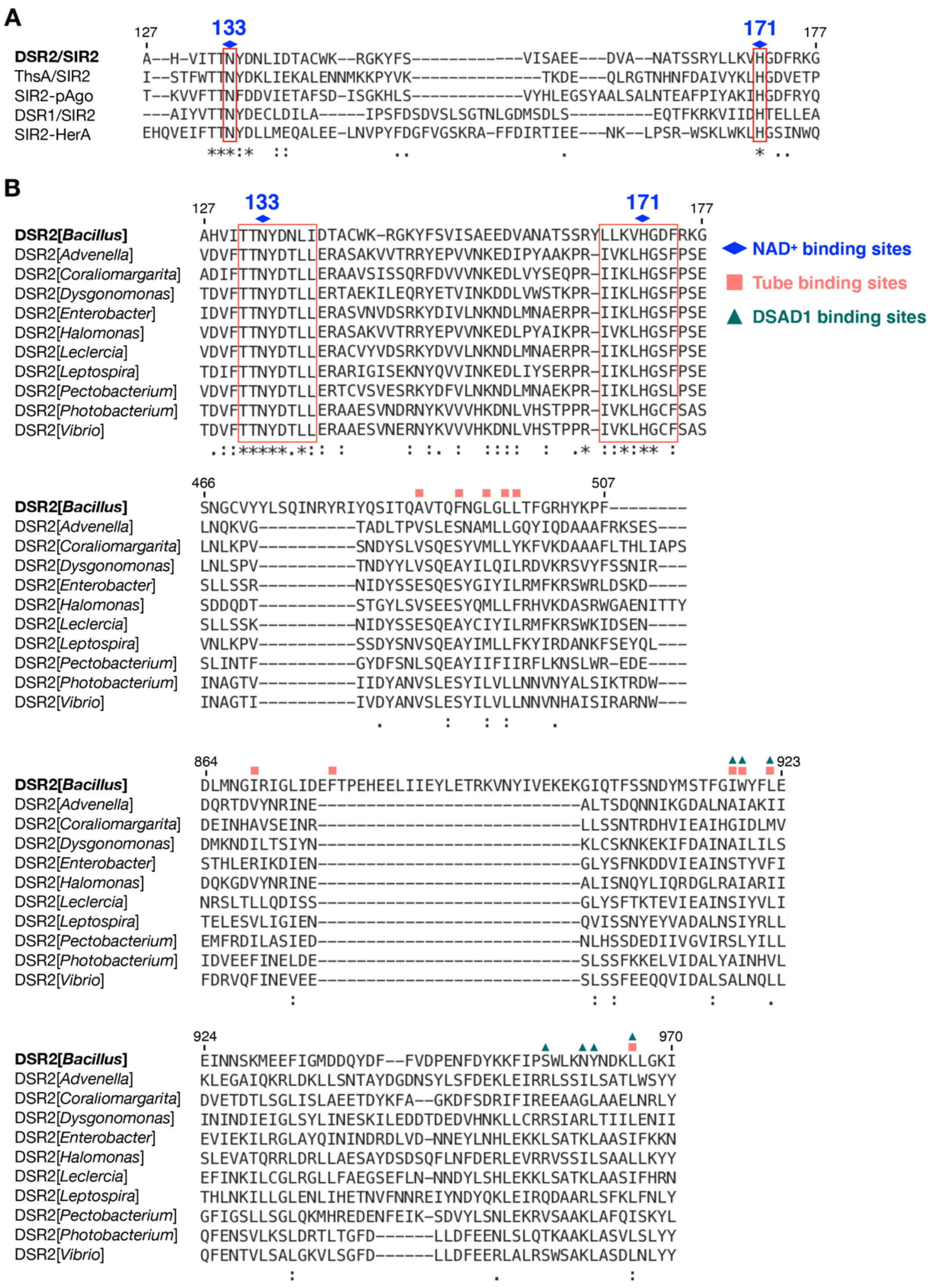

Supplementary Figure 6. Multiple sequence alignment of sirtuin proteins and DSR2 from different species. A. Sequence alignment of the NAD$^+$-binding region of DSR2, ThsA, pAgo, DSR1 and HerA. Residues Asn133 and His171 of DSR2 are shown in the red frame. **B.** Sequence alignment of DSR2 from different species. The blue diamond represents the NAD$^+$ binding sites, the salmon-colored square represents the Tube binding sites, and the teal triangle represents the DSAD1 binding sites.

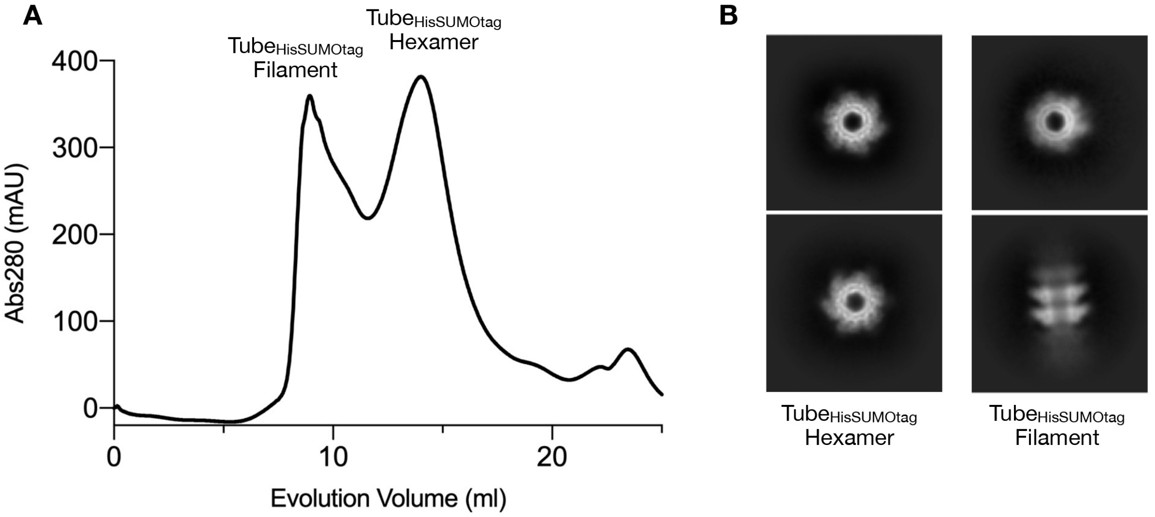

Supplementary Figure 7. Phage tail tube protein forms a supramolecular complex that fails to bind DSR2. A. Size-exclusion chromatography analysis of recombinant Tube with His6-SUMO-tag, peak fraction collected to prepare samples for cryo-EM. **B.** Representative reference-free 2D-class averages of Tube. Hexamers of Tube form a wreath-like shape. Hexamers are then stacked in an orderly manner to form a filament.

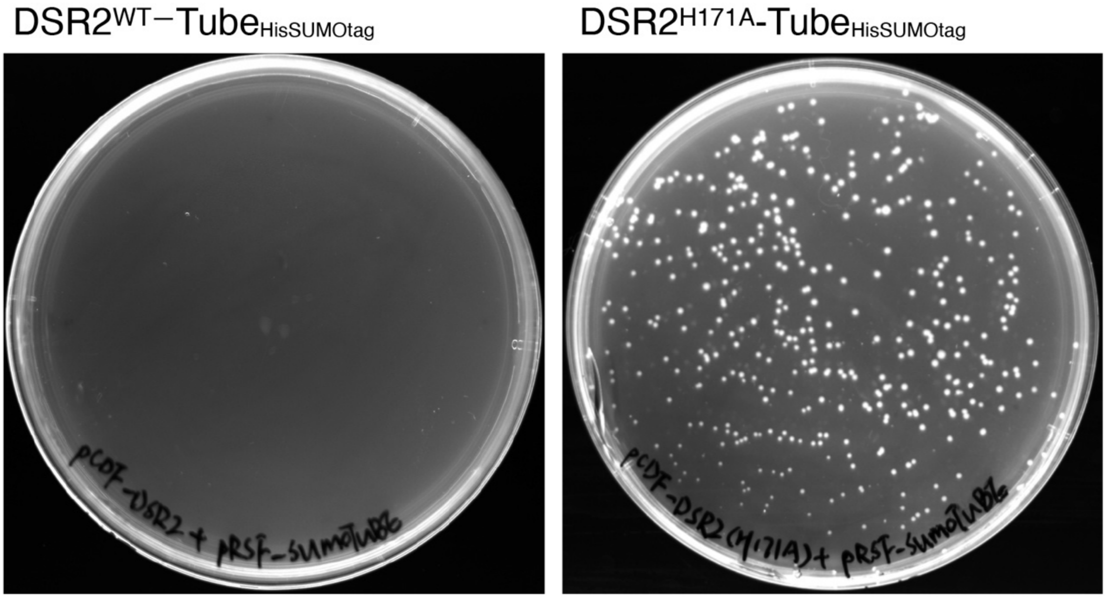

Supplementary Figure 8. DSR2 Expression of wild-type DSR2 with Tube protein is toxic to cells (left). DSR2 in this experiment was mutated (H171A) to avoid toxicity (right).

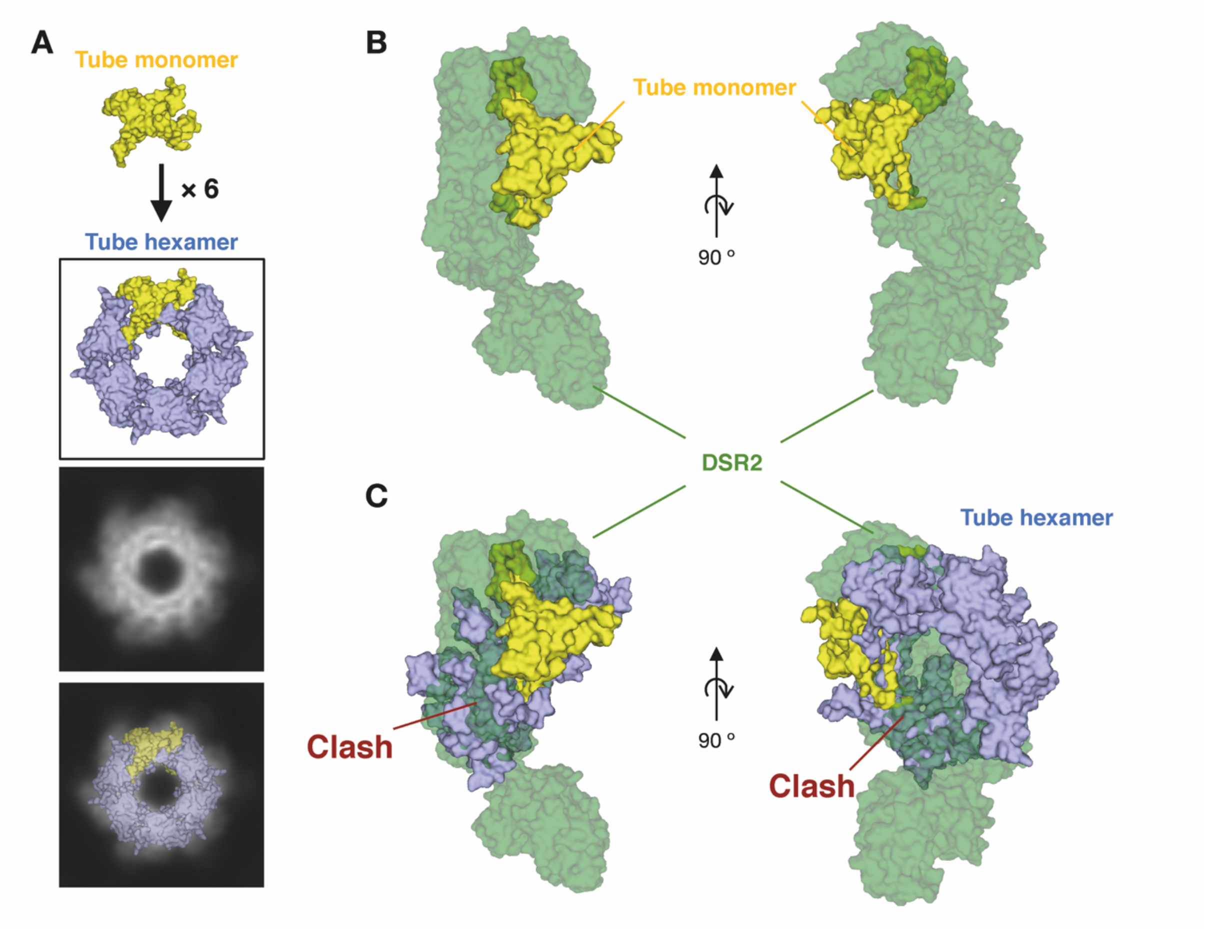

Supplementary Figure 9. Structure superimposition of hexameric Tube with DSR2-Tube complex. A. Atomic structure of monomeric and hexameric Tube. The monomeric Tube assembles into hexameric rings. Representative cryo-EM 2D class averages of the Tube hexamer; cryo-EM 2D class averages of the Tube hexamer superimposed on its atomic structure. **B.** Front and side views of the DSR2-Tube complex. **C.** Structural alignment of the Tube hexamer (light blue) and Tube monomer (yellow). There is a steric clash between Tube hexamer and DSR2 (green).

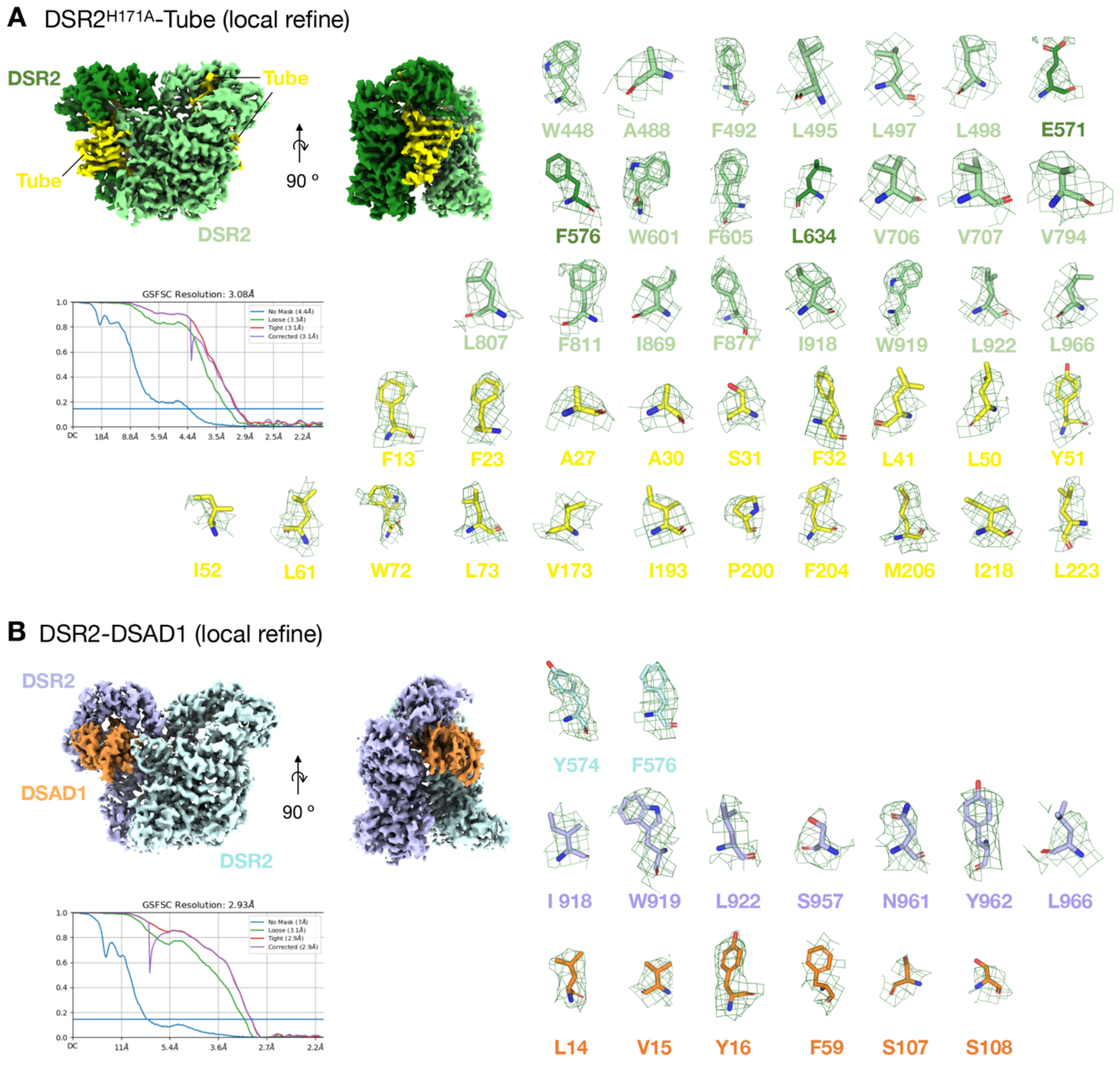

Supplementary Figure 10. Local resolutions of the DSR2^{H171A}-Tube (A) and DSR2-DSAD1 (B). Cryo-EM densities for the key interacting residues are shown.

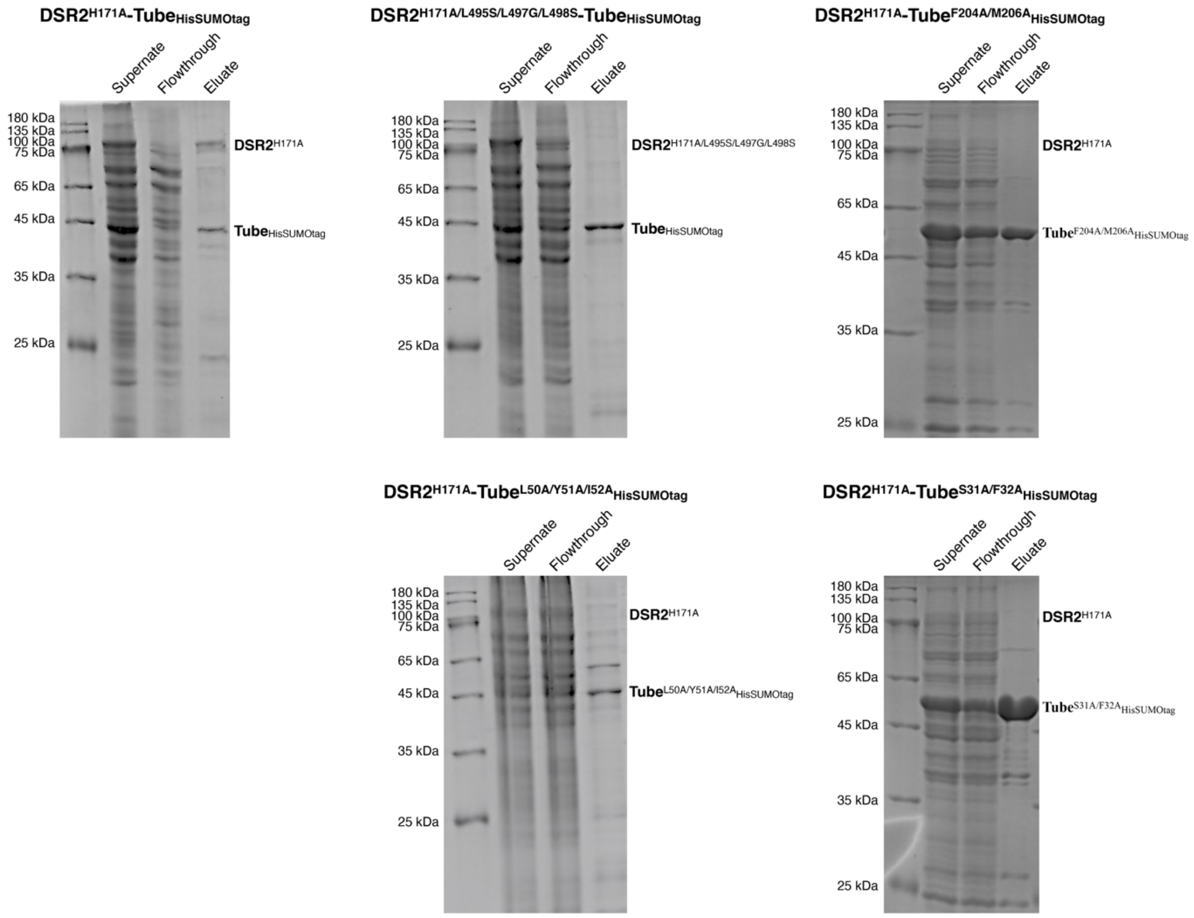

Supplementary Figure 11. *In vivo* pull-down assays of the DSR2 and Tube proteins. DSR2^{H171A} without tag and Tube protein with His6-SUMO tag were coexpressed in *E. coli* and purified with Ni column to obtain the DSR2^{H171A}-Tube complex (top left). The DSR2 mutant H171A/L495S/L497G/L498S (top middle) fails to form a complex with Tube. The Tube mutants L50A/Y51A/I52A (bottom left), F204A/M206A (top right), and S31A/F32A (bottom right), are unable to pull down DSR2^{H171A}.

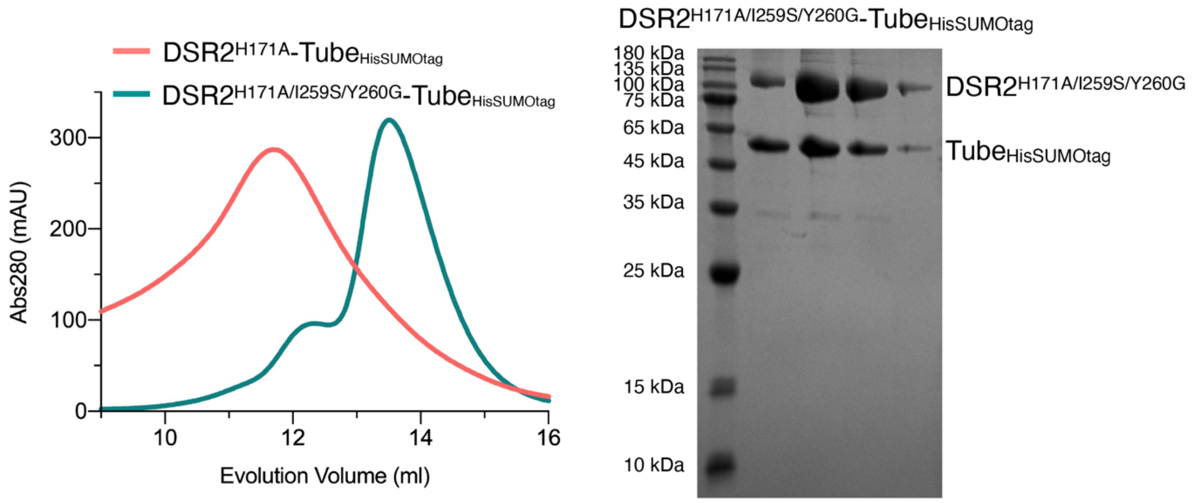

Supplementary Figure 12. Size exclusion chromatography analysis of DSR2^{H171A}-Tube$_{HisSUMOtag}$ and DSR2$^{H171A/I259S/Y260G}$-Tube$_{HisSUMOtag}$. The peak position shifted to the rear indicates that the DSR2$^{I259S/Y260G}$ mutant is dimerized.

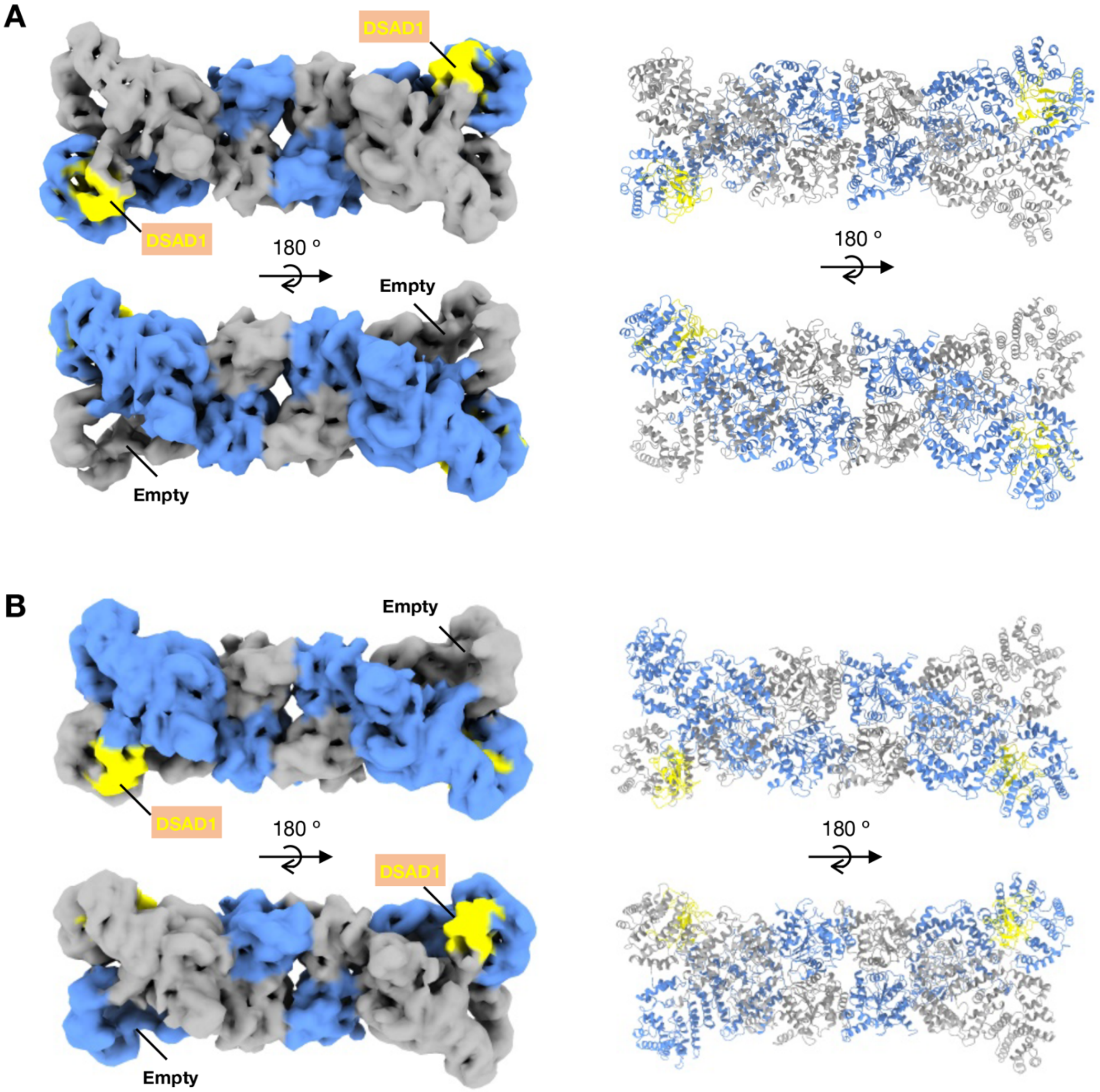

Supplementary Figure 13. Two binding modes of the DSR2-DSAD1 complex. A. Cryo-EM density map (left) and the structure (right) of DSR2 combined with DSAD1 bound on diagonal side in two views. **B**. Cryo-EM density map (left) and the structure (right) of DSR2 combined with DSAD1 bound on the same side in two views. DSR2 tetramer are shown in grey and blue, DSAD1 were shown in yellow.

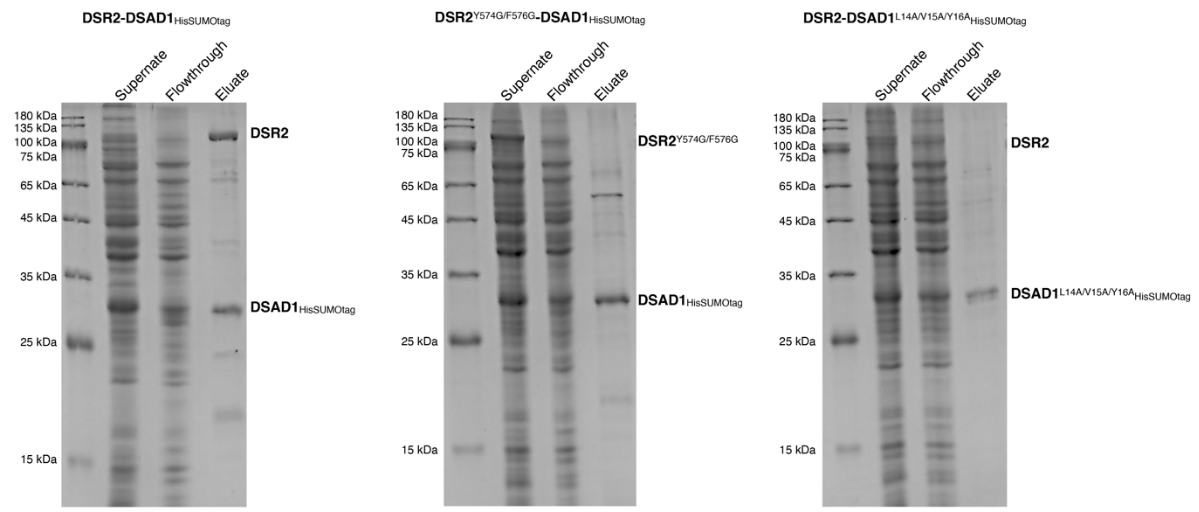

Supplementary Figure 14. *In vivo* pull-down assays of the DSR2 and DSAD1. DSR2 without tag and DSAD1 protein with His6-SUMO tag were co-expressed in *E. coli* and purified with Ni-column to obtain the DSR2-DSAD1 complex (left). The DSR2 mutant Y574G/F576G did not interact with DSAD1 (middle). DSAD1 with point mutations (L14A/V15A/Y16A) also lost the ability to interact with DSR2 (right).

Supplementary Table 1. Cryo-EM data collection, refinement and validation statistics

	DSR2 Apo<br>PDB: 8WKX<br>EMDB: 37610	DSR2^{H171A}-Tube<br>PDB: 8WKS<br>EMDB: 37606	DSR2-DSAD1<br>PDB: 8WKT<br>EMDB: 37607
**Data collection processing**			
Magnification	130,000	130,000	130,000
Voltage (kV)	300	200	300
Electron exposure (e−/Å^2)	60	60	60
Defocus rang (μm)	-1.2 ~ -1.8	-1.2 ~ -1.8	-1.2 ~ -1.8
Pixel size (Å)	1.07	1.0	1.04
Symmetry imposed	C1	C1	C1
Initial particle images (no.)	457,033	943,799	629,718
Final particle images (no.)	80,223	127,766	105,782
Map resolution (Å)	4.15	3.58	3.49
FSC threshold	0.143	0.143	0.143
Map resolution range (Å)	-	-	-
**Refinement**			
Initial model used (PDB code)	None	None	None
Model resolution (Å)	4.5	4.1	3.8
FSC threshold	0.5	0.5	0.5
Model resolution range (Å)	-	-	-
Map sharpening *B* factor (Å)	-114.1	-131.2	-121
Model composition			
Non-hydrogen atoms	16599	36919	21944
Protein residues	1993	4459	2644
B factor (Å^2)			
Protein	60.03	61.08	93.01
R.m.s. deviations			
Bond lengths (Å)	0.002	0.002	0.04
Bond angles (°)	0.581	0.567	0.614
Validation			
MolProbity score	1.87	2.01	1.81
Clashscore	10.80	15.15	9.89
Poor rotamers (%)	0.00	0.22	0.5
Ramachandran plot			
Favored (%)	95.47	95.31	95.78
Allowed (%)	4.53	4.69	4.22
Disallowed (%)	0	0	0

Supplementary Table 2. Oligonucleotides used in this work.

Oligonucleotide	Sequence（5'-3' direction）	Description
DSR2-F	ACCATCATCACCACAGCCAGATGGTGAAGGTTGATCTGGAGAGCAA	Forward primer for the amplification of the DSR2 gene
DSR2-R	GCAGCGGTTTCTTTACCAGACTTAGATAAAGTAGTTCATCAGGATCTCCAG	Reverse primer for the amplification of the DSR2 gene
Tube-F	AGAGAACAGATTGGTGGATCCATGAAGACCGTTATTCAGGA	Forward primer for the amplification of the TUBE gene
Tube-R	CGACTTAAGCATTATGCGGCCGCTTATTCTGTGGTACCTCCAAGG	Reverse primer for the amplification of the TUBE gene
DSAD-F	AGAGAACAGATTGGTGGATCCATGATCGAAATCTTCAAGGA	Forward primer for the amplification of the DSAD1 gene
DSAD-R	CGACTTAAGCATTATGCGGCCGCTTAATCCAGATAAACCACTTCTT	Reverse primer for the amplification of the DSAD1 gene
171-F	CAGATATCTGCTGAAGGTCGCCGGAGATTTTCGTAAAGGCT	Mutagenic primer (top strand) for the H171A mutation.
171-R	CTTTACGAAAATCTCCGGCGACCTTCAGCAGATATCTGCTGCT	Mutagenic primer (boottom strand) for the H171A mutation.
259/260-F	CCGATTGAGAACGAAACCCTGTCTGGTTATGAAAATAAGGGCCTGCGTA	Mutagenic primer (top strand) for the I259S/Y260G mutation.
259/260-R	CGCAGGCCCTTATTTTCATAACCAGACAGGGTTTCGTTCTCAATCGGTG	Mutagenic primer (boottom strand) for the I259S/Y260G mutation.
143/148-F	GTGGGAAACGTGGTAAGGGTTTTAGCGTAATTAGCGCAGAAGAAG	Mutagenic primer (top strand) for the W143G/Y148G/Y552G/V556S/F559G/N563S mutation.
143/148-R	AAAACCCTTACCACGTTTCCCACATGCGGTGTCAATCAGATT	Mutagenic primer (bottom strand) for the W143G/Y148G/Y552G/V556S/F559G/N563S mutation.
552/556/559/563-F	GGCGATGACACATCTAAGCTGGGCGAACTGACCTCCAAAGTTCGTTCCGAAATGAG	Mutagenic primer (top strand) for the W143G/Y148G/Y552G/V556S/F559G/N563S mutation.
552/556/559/563-R	GGAGGTCAGTTCGCCCAGCTTAGATGTGTCATCGCCTAAAAACTGATTGTCGCTAA	Mutagenic primer (bottom strand) for the W143G/Y148G/Y552G/V556S/F559G/N563S mutation.
204/206-F	GGCGAAGCTGAAGCGAGCCTGGAAAATGGGAATGC	Mutagenic primer (top strand) for the F204A/M206A mutation.
204/206-R	GGCTCGCTTCAGCTTCGCCGGACGGAGAAACATTCG	Mutagenic primer (bottom strand) for the F204A/M206A mutation.
495/497/498-F	GGTTCAGGTGGATCTACCTTTGGTCGTCATTATAAACCGT	Mutagenic primer (top strand) for the L495S/L497G/L498S mutation.
495/497/498-R	GACCAAAGGTAGATCCACCTGAACCGTTAAACTGGGT	Mutagenic primer (bottom strand) for the L495S/L497G/L498S mutation.
31/32-F	AGACCGCAGCTGCTAGCCAGGCGATTAGCGAAGAAA	Mutagenic primer (top strand) for the S31A/F32A mutation.
31/32-R	CTGGCTAGCAGCTGCGGTCTGTGCTTCGGCGGTG	Mutagenic primer (bottom strand) for the S31A/F32A mutation.
50/51/52-F	TAAACCGGCGGCCGCCCTGAAAAGCGAAAAAGAAATCAATC	Mutagenic primer (top strand) for the L50A/Y50A/I52A mutation.
50/51/52-R	CTTTTCAGGGCGGCCGCCGGTTTATTTCCAATGCCTC	Mutagenic primer (bottom strand) for the L50A/Y50A/I52A mutation.
14/15/16-F	CCCACGACGCGGCTGCTCACAGCAAAATTAACACC	Mutagenic primer (top strand) for the L14A/V15A/Y16A mutation.
14/15/16-R	GCTGTGAGCAGCCGCGTCGTGGGTGGCGCCGGT	Mutagenic primer (bottom strand) for the L14A/V15A/Y16A mutation.
574/576-F	GGTAGCGGTAGCGGCGGTATGAGCAGCGATATAGTTGTTTTAC	Mutagenic primer (top strand) for the Y574G/F576G mutation.
574/576-R	CATACCGCCGCTACCGCTACCTTCGCTCATTTCGGAACGAAC	Mutagenic primer (bottom strand) for the Y574G/F576G mutation.
463/471-F	GACGAAAGCAATGGATGTGTTGGGTATTTAAGCCAGATTAATCGTTACCG	Mutagenic primer (top strand) for the Y574G/F576G mutation.
463/471-R	AACACATCCATTGCTTTCGTCTCCGCTGTTAAGGATAATATTGCTATAC	Mutagenic primer (bottom strand) for the I463G/Y471G mutation.
521/522/531/532-F	ATGATCTGTTCAACGGTGGGGGATTTGAATTTCAGAAAAAGTATAAGATCCTG	Mutagenic primer (top strand) for the N521G/F522G/M531G/P532G mutation.
521/522/531/532-R	CCCACCGTTGAACAGATCATCGATATTGCCACCGGTCATTTCACGTTCAATGC	Mutagenic primer (bottom strand) for the N521G/F522G/M531G/P532G mutation.
143-148-F	GTGGAAGCAGTGGCTCAAGTTTTAGCGTAATTAGCGCAGA	Mutagenic primer (top strand) for the W143G/K144S/R145S/G146/K147S/Y148S mutation.
143-148-R	ACTTGAGCCACTGCTTCCACATGCGGTGTCAATCAGAT	Mutagenic primer (bottom strand) for the W143G/K144S/R145S/G146/K147S/Y148S mutation.
299-308-F	TCTGGATCTGGCTCGGGGTCTGGTTCGATTGATTATATTTACGGGAAAAT	Mutagenic primer (top strand) for the N299G/K300S/F301G/I302S/T303G/K304S/D305G/D306S/E307G/V308S mutation.
299-308-R	AGACCCCGAGCCAGATCCAGACCCCTCTTGTGATTCGATCAGCAGAT	Mutagenic primer (bottom strand) for the N299G/K300S/F301G/I302S/T303G/K304S/D305G/D306S/E307G/V308S mutation.
