# Supplementary material for: Molecular basis of bacterial DSR2 anti-phage defense and viral immune evasion
Source: Nat Commun. 2024 May 10;15:3954. doi: 10.1038/s41467-024-48291-4 (PMC11087589; doi:10.1038/s41467-024-48291-4)
Supplement: Supplementary file 3 — Description of Additional Supplementary Files [file 41467_2024_48291_MOESM3_ESM.pdf]

File name: Supplementary Movie 1
Description: Conformational changes of DSR2 upon binding to the Tube protein.
