# Supplementary material for: Molecular basis of bacterial DSR2 anti-phage defense and viral immune evasion
Source: Nat Commun. 2024 May 10;15:3954. doi: 10.1038/s41467-024-48291-4 (PMC11087589; doi:10.1038/s41467-024-48291-4)
Supplement: Supplementary file 5 — Reporting Summary [file 41467_2024_48291_MOESM5_ESM.pdf]

Corresponding author(s): Ang Gao, Jing Yang, Jiafeng Huang

Last updated by author(s): Apr 7, 2024

# Reporting Summary

Nature Portfolio wishes to improve the reproducibility of the work that we publish. This form provides structure for consistency and transparency in reporting. For further information on Nature Portfolio policies, see our Editorial Policies and the Editorial Policy Checklist.

## Statistics

For all statistical analyses, confirm that the following items are present in the figure legend, table legend, main text, or Methods section.

| n/a | Confirmed | |
|---|---|---|
| ☐ | ☒ | The exact sample size (*n*) for each experimental group/condition, given as a discrete number and unit of measurement |
| ☐ | ☒ | A statement on whether measurements were taken from distinct samples or whether the same sample was measured repeatedly |
| ☒ | ☐ | The statistical test(s) used AND whether they are one- or two-sided<br>*Only common tests should be described solely by name; describe more complex techniques in the Methods section.* |
| ☒ | ☐ | A description of all covariates tested |
| ☒ | ☐ | A description of any assumptions or corrections, such as tests of normality and adjustment for multiple comparisons |
| ☐ | ☒ | A full description of the statistical parameters including central tendency (e.g. means) or other basic estimates (e.g. regression coefficient) AND variation (e.g. standard deviation) or associated estimates of uncertainty (e.g. confidence intervals) |
| ☒ | ☐ | For null hypothesis testing, the test statistic (e.g. *F*, *t*, *r*) with confidence intervals, effect sizes, degrees of freedom and *P* value noted<br>*Give P values as exact values whenever suitable.* |
| ☒ | ☐ | For Bayesian analysis, information on the choice of priors and Markov chain Monte Carlo settings |
| ☒ | ☐ | For hierarchical and complex designs, identification of the appropriate level for tests and full reporting of outcomes |
| ☒ | ☐ | Estimates of effect sizes (e.g. Cohen's *d*, Pearson's *r*), indicating how they were calculated |

*Our web collection on statistics for biologists contains articles on many of the points above.*

## Software and code

Policy information about availability of computer code

| | |
|---|---|
| Data collection | SerialEM 3.8 |
| Data analysis | MotionCor 2 1.3.2, Cryosparc v4.3.1, Chimera 1.10.1, ChimeraX 1.6.1, Phenix 1.19.1, Coot 0.9.6, Pymol2.5, ImageJ 2.14.0, Prism v8.4.3 |

For manuscripts utilizing custom algorithms or software that are central to the research but not yet described in published literature, software must be made available to editors and reviewers. We strongly encourage code deposition in a community repository (e.g. GitHub). See the Nature Portfolio guidelines for submitting code & software for further information.

## Data

Policy information about availability of data

All manuscripts must include a data availability statement. This statement should provide the following information, where applicable:

- Accession codes, unique identifiers, or web links for publicly available datasets
- A description of any restrictions on data availability
- For clinical datasets or third party data, please ensure that the statement adheres to our policy

The cryo-EM density maps have been deposited into the Electron Microscopy Data Bank (EMDB) under accession numbers EMD-37610 [https://www.ebi.ac.uk/emdb/EMD-37610] (apo DSR2 tetramer), EMD-37606 [https://www.ebi.ac.uk/emdb/EMD-37606] (DSR2H171A-Tube), and EMD-37607 [https://www.ebi.ac.uk/emdb/EMD-37607] (DSR2-DSAD1). The atomic coordinates have been deposited at the Protein Data Bank (PDB) under accession numbers 8WKX [https://www.rcsb.org/structure/unreleased/8WKX] (apo DSR2 tetramer), 8WKS [https://www.rcsb.org/structure/unreleased/8WKS] (DSR2H171A-Tube), and 8WKT

[https://www.rcsb.org/structure/unreleased/8WKT] (DSR2-DSAD1). The source data underlying Figs. 2A, 4D, 5I and 6D and Supplementary Figs. 1, 5, 7, 11,12 and 14 are provided as a Source Data file. Other data are available from the corresponding author upon reasonable request.

## Research involving human participants, their data, or biological material

Policy information about studies with human participants or human data. See also policy information about sex, gender (identity/presentation), and sexual orientation and race, ethnicity and racism.

| | |
|---|---|
| Reporting on sex and gender | N/A |
| Reporting on race, ethnicity, or other socially relevant groupings | N/A |
| Population characteristics | N/A |
| Recruitment | N/A |
| Ethics oversight | N/A |

Note that full information on the approval of the study protocol must also be provided in the manuscript.

# Field-specific reporting

Please select the one below that is the best fit for your research. If you are not sure, read the appropriate sections before making your selection.

☒ Life sciences ☐ Behavioural & social sciences ☐ Ecological, evolutionary & environmental sciences

For a reference copy of the document with all sections, see nature.com/documents/nr-reporting-summary-flat.pdf

# Life sciences study design

All studies must disclose on these points even when the disclosure is negative.

| | |
|---|---|
| Sample size | No statistical methods were used to predetermine sample size. The data size for cryoEM was determined by the availability of the microscope time and the particle density on the grids. Sufficient cryoEM data were collected to achieve the reported resolution of map, which is sufficient for model building. |
| Data exclusions | No data were excluded. |
| Replication | Data in the enzyme activity assays are presented as mean +/- s.e.m. for five independent experiments. |
| Randomization | No group allocation was needed for functional experiments in this study. |
| Blinding | The study was not blinded since visual inspection of both cryoEM reconstructions and other experiments was necessary to determine the quality of the data. |

# Reporting for specific materials, systems and methods

We require information from authors about some types of materials, experimental systems and methods used in many studies. Here, indicate whether each material, system or method listed is relevant to your study. If you are not sure if a list item applies to your research, read the appropriate section before selecting a response.

## Materials & experimental systems

| n/a | Involved in the study |
|---|---|
| ☒ | ☐ Antibodies |
| ☒ | ☐ Eukaryotic cell lines |
| ☒ | ☐ Palaeontology and archaeology |
| ☒ | ☐ Animals and other organisms |
| ☒ | ☐ Clinical data |
| ☒ | ☐ Dual use research of concern |
| ☒ | ☐ Plants |

## Methods

| n/a | Involved in the study |
|---|---|
| ☒ | ☐ ChIP-seq |
| ☒ | ☐ Flow cytometry |
| ☒ | ☐ MRI-based neuroimaging |
